# It’s a trap: Optimizing detection of rare small mammals

**DOI:** 10.1371/journal.pone.0213201

**Published:** 2019-03-05

**Authors:** Kristina M. Harkins, Doug Keinath, Merav Ben-David

**Affiliations:** 1 Department of Zoology and Physiology and Program in Ecology, University of Wyoming, Laramie, Wyoming, United States of America; 2 U. S. Fish and Wildlife Service, Cheyenne, Wyoming, United States of America; Sichuan University, CHINA

## Abstract

Improving detection probabilities for rare species is critical when assessing presence or habitat associations. Our goal was to create a new small mammal trapping protocol that improved detection of rare species, such as the olive-backed pocket mouse (*Perognathus fasciatus*). We used three trap and bait types and trapped an area 4.4 times larger than the standard grid. We also assessed the effect of captures of non-target species on detection probability of pocket mice. Regardless of species, trap success was higher for Havaharts. We found that bait and trap type selection varied significantly by species, with pocket mice showing strongest selection for Havahart traps baited with bird seed. Increasing grid size, while maintaining a similar trapping effort, resulted in higher detection probability, although our analyses showed that effective grids can be about three-quarters of the size we use to achieve similar results. We were also able to demonstrate that by deploying a combination of different traps and baits it is possible to overcome the potential effect of non-target species (e.g., deer mice, *Peromyscus maniculatus*) on the detection probability of pocket mice. Our results show that simple changes to standard small-mammal trapping methods can dramatically increase the detectability of rare and elusive small mammals. Increasing detection probability of rare components of a community can improve the results and understanding of future studies.

## Introduction

Rapid and global environmental change increases the need to identify and conserve critical habitats for at-risk and endangered species [1]. Identifying such critical habitats often relies on occupancy modeling, which has become a common tool to monitor wildlife populations when insufficient captures prevent the use of mark-recapture analysis or when individual identification is impossible [2]. Occupancy modeling is a logistic regression-based framework that estimates the probability of a species occupying sampled sites (occupancy) while accounting for the probability of detecting the species using the given sampling methods (detectability). Imperfect detection is a frequent sampling problem when animals are rare, populations are small, or individuals are difficult to observe [3,4]. However, even when species are fairly easy to observe and identify, detection is often imperfect [5,6]. Survey methods and conditions can alter the behavior of animals or affect surveyor performance, further reducing the ability to distinguish between a true negative, where a species is absent from the survey area, and a false negative, when a species is present but undetected during the survey [2,7]. Including estimates of detectability in occupancy analyses is crucial because false negatives can lead to a biased and imprecise estimation of habitat characteristics and their relative influence on the occurrence of the target species [8,9]. Accounting for detection probability increases the performance of models estimating occupancy [4], especially for rare or difficult to detect species [10,11]. As detection probability increases, estimated occupancy becomes more precise (i.e. tighter confidence intervals; [2]). In turn, that precision directly increases our ability to track temporal changes in occupancy in surveyed areas and create refined habitat suitability models. Both these results translate into better ecological understanding of the systems in question and, consequently, an improved ability to make effective management decisions.

Rare species generally require specific survey methods to optimize their detection. Over the last few decades, biologists developed new techniques and used advanced technology to increase detectability for a variety of rare and elusive species, such as scat detection dogs for North American forest carnivores [12], camera traps for terrestrial rainforest mammals [13,14], drones for Florida manatees (*Trichechus manatus latirostris*; [15]), eDNA for amphibians and other aquatic species [7,16], and infrared imagery for bats and polar bears (*Ursus maritimus*, [17]). For small mammals, however, the most accurate form of detection remains standardized trapping.

The assumption of equal detection probability may be invalid even for individuals of the same species based on differences such as sex, age, body condition [18] or behavior [19]. Similarly, it is likely that detection probability differs among species. Many studies have evaluated the effect of trap type on capture rates or detection of small mammals [20–27], and a select few have examined the efficiency of both trap and bait types [22,25,26]. However, most of these studies addressed questions related to species richness and community composition rather than attempting to assess the factors affecting the occupancy of rare species. While these comparisons are valuable, many small mammal researchers are interested in drawing conclusions for, and thus optimizing the detection of, specific rare species. Rare species have gained increased interest in recent years because, given the current level of accelerated development and global climate change, such species are at greater risk of population declines and extinctions [28,29]. Furthermore, tests of ecological theories such as island biogeography, intraspecific competition and the influence of ecosystem productivity on species diversity [30–32], using small mammal communities as models, may be invalid because of potential bias associated with the detection of rare or low abundance species [33].

The majority of small mammal studies use Sherman live traps baited with a mix of peanut butter, oats and occasionally molasses. These traps are typically set in a 10×10 trapping grid with 10 m spacing, which results in a realized sampling area of 0.81 hectares [24,34–36]. When trapping granivorous rodents, some researchers have used seeds as bait (e.g., mixed bird seed and rolled oats), but the effectiveness of this relative to more standard bait types has not been formally quantified [37–39].

As part of a larger study focused on clarifying the distribution and habitat use of rare pocket mice (*Perognathus* spp.) across the sagebrush-dominated basins of Wyoming, we tested a new trapping protocol designed to maximize capture success and assess the effects of community composition on detection probability of these small mammals. We addressed three primary questions. First, do pocket mice prefer specific combinations of trap and bait relative to other members of the small mammal community in which they occur? Second, is the detection of pocket mice influenced by trapping protocols, specifically trap type, bait types and size of trapping grids? Third, how do captures of non-target species affect detection probability of pocket mice?

## Materials and methods

### Study area

**Fig 1:**
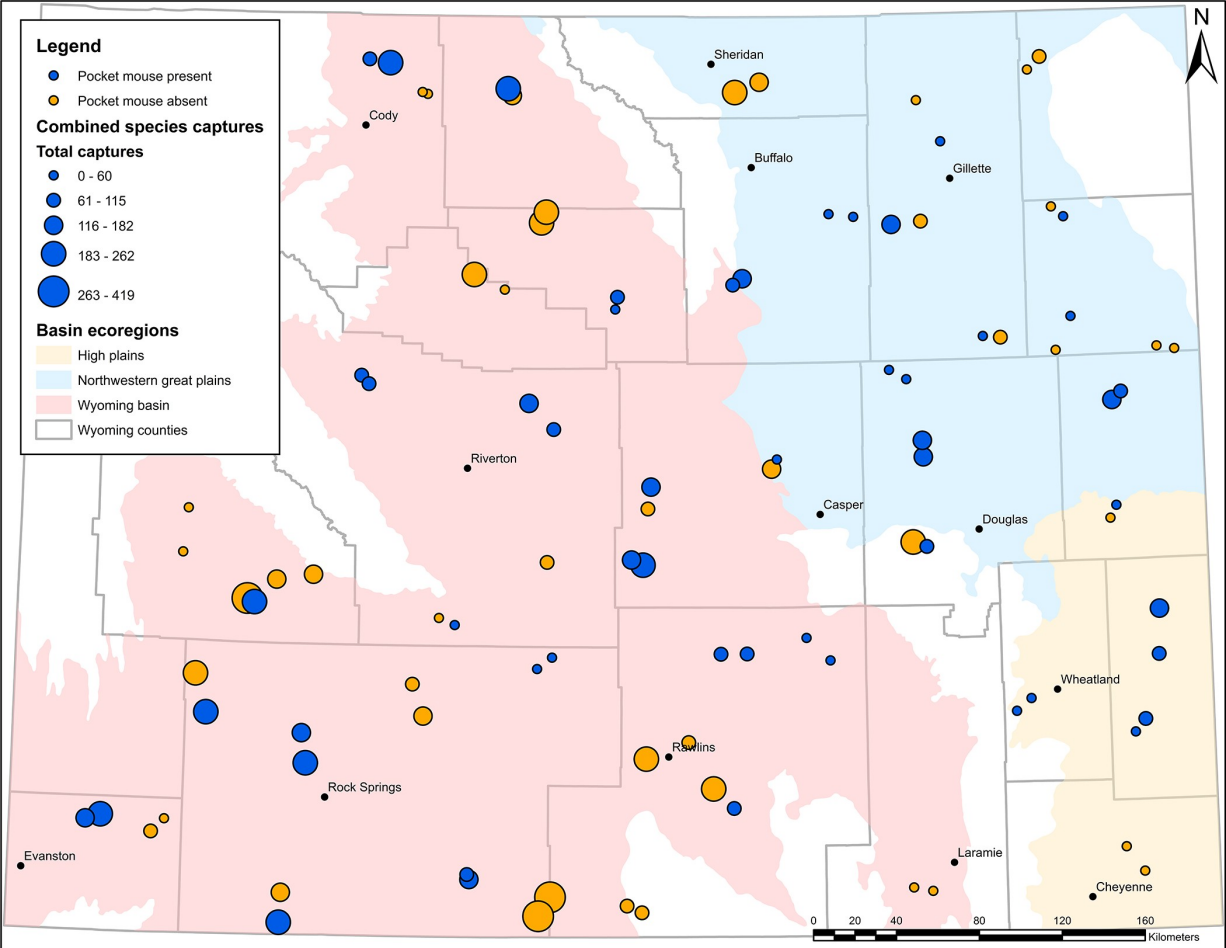
Trapping locations for both 2015 and 2016 across Wyoming sagebrush shrubland and prairie grassland. Total captures for all species shown in different sizes. Trapping sites where pocket mice were present are in blue. Sites where pocket mice were not detected are in orange.

Our study area included all sagebrush shrublands and prairie grasslands in the state of Wyoming (186,025 km^2^; Fig 1). These habitats receive a minimum of 15 cm and a maximum of 51 cm of precipitation per year [40] and experience an average temperature range of 0° C in winter to 15.5° C in summer [41]. Big sagebrush (*Artemisia tridentata*) is a keystone plant in sagebrush shrubland ecosystems, while prairie grasslands are dominated by a variety of species including blue grama (*Bouteloua gracilis*), needle-and-thread (*Stipa comata*), wheatgrasses (*Elymus* and *Pascopyrum*) and Sandberg’s bluegrass (*Poa secunda*). Non-native grasses include cheat grass (*Bromus tectorum*) and smooth brome (*Bromus inermis*). These ecosystems support a large number of species including pronghorn (*Antilocapra americana*), greater sage-grouse (*Centrocercus urophasianus*), prairie dogs (*Cynomys* ssp.), large grazing ungulates, and a variety of grassland and sagebrush obligate songbirds such as sage thrasher (*Oreoscoptes montanus*), Brewer’s sparrow (*Spizella breweri*), and McCown’s longspur (*Rhynchophanes mccownii*; [40,42]. Most small mammal species in sagebrush shrublands and prairie grasslands, including deer mice (*Peromyscus*), voles (*Microtus* and *Lemmiscus*), pocket mice (*Chaetodipus* and *Perognathus*), kangaroo rats (*Dipodomys*), chipmunks (*Tamias*), and ground squirrels (*Urocitellus* and *Ictidomys*), obtain needed fluids primarily from food rather than drinking water [43,44].

### Site selection

We surveyed 47 sites in 2015 and 52 sites in 2016 for a total of 99 locations over the course of the study. We selected sampling locations on public lands throughout Wyoming at or below 2300 m in elevation [45] with less than 30% tree canopy cover [46]. We stratified the study area by the three basin ecoregions: High Plains, Northwest Great Plains and Wyoming Basin [47]. This stratification was needed due to substantial differences in the extent of public land among these ecoregions, which resulted in an uneven sampling effort when points were generated randomly. Within each ecoregion, initial survey sites were placed across the landscape in a spatially-balanced random fashion using the GRTS package in program R [48]. Following initial site selection, we added a second sampling location placed randomly within a 25 km buffer area, but greater than 5 km from the initial site. Logistically, these additional locations could be trapped concurrently with the initial sites but were sufficiently distant as to represent independent sampling areas. This approach allowed us to maximize survey efforts. Each site was surveyed following the single-season occupancy modeling framework [2].

### Small mammal trapping

To maximize the probability of encountering pocket mice within each sampling area we set traps in a rectangular grid of 4×20 points. Grid points were spaced at 25 m intervals, resulting in a 3.56 hectare trapping area. At each grid point we placed one Havahart (HA; Woodstream Corporation, Lititz, PA) and one Sherman (SH; H. B. Sherman Traps, Inc, Tallahassee, FL) live traps near each other (<0.5 m). Additionally, for trapping grids surveyed in 2016 one trap line also included a Longworth trap (LW; NHBS Ltd, Devon, UK) at each grid point. Trapping effort was lower for Longworths because we had fewer of these traps available.

We attracted small mammals to our traps with three different bait types: peanut butter mixed with oats (PB), 3-way horse feed with molasses (HF; Profile Horse Feeds: 3-Way Grain Mix, Fort Collins, CO) and sterilized (slowly cooked at 50°C for 6 hours) bird seed mix (BS) consisting of 4% black oil sunflower, 15% millet, 42% milo, 30% cracked corn, 7% wheat and 2% calcium carbonate (Ace Wild Bird Seed, Ace Hardware Corporation, Oak Brook, IL). Bait was systematically alternated among grid points, such that all traps at each point were baited with the same type but flanked by different bait on all sides. Additionally, we provided a ball of polyester fiber-filling in each trap for animals to use as bedding. We baited and opened traps each evening within three hours of sunset. Traps were closed and animals were processed the following morning within three hours of sunrise. Each site was trapped for 4 consecutive nights unless a severe weather event prevented the opening of traps.

Trapped animals were individually marked using passive integrated transponder tags (PIT tags; Biomark, Boise ID) or numbered ear tags, depending on ear size, identified to species and sexed. We measured body length, tail length, ear length, hind foot length (to the nearest 0.1 cm), and weight (nearest 0.1 g) for our target genera (*Chaetodipus*, *Lemmiscus*, and *Pergonathus*). Additionally, we clipped a small patch of hair above the tail of each individual to readily identify recaptures. We recorded the trap type, bait type and grid point for each capture. All animals were released at the site of their original capture. This study followed accepted, humane trapping methods [49] and was approved by an Independent Animal Care and Use Committee (IACUC) at the University of Wyoming. The level of mortality was reported to the IACUC as part of its annual review and procedures were re-approved by the committee.

### Data analysis

To test for selection of trap and bait by species, we employed a compositional analysis of use with the trap and bait combinations coded as “habitat” types for each species [50]. Compositional analysis is conducted in two steps. First a Wilks lambda value is used to determine whether habitat selection occurs. Following evidence of selection, a ranking matrix is constructed to identify the specific habitat types (or in our case particular bait-trap combinations) that are used disproportionally relative to their availability [50]. To account for inter-site variability, the used and available trap and bait combinations were calculated for each site and were treated as the individuals in the compositional analysis. We conducted a compositional analysis for all captures combined and then separately for each of the 10 species with a sufficient number of captures. Due to the unequal trapping effort of Longworth traps, we excluded them from the overall compositional analysis and analyzed them separately based only on grid points where they were present.

We used the R package *Unmarked* [51] to model the detection probabilities of pocket mice. To test the effect of trap type, we considered each independently. For example, when considering HA traps, we recorded pocket mice as present at a site only if they were trapped in a HA; they were otherwise considered absent for the purposes of this analysis even if they were caught in SH traps. For each type of trap or bait, we ran a ‘null’ model where detection was constant across trap nights and a second model where detection differed temporally by night. We ran both of these model types to assess the overall improvement in detection probability between different trap and bait types and the possible nightly improvement in detection for desirable trap and bait types. To simplify these analyses, we excluded all other potential covariates. We used Akaike Information criteria for small sample size (AICc; [52]) to identify the best fit model.

To fully assess the effect of bait and trap choices on detection probability of pocket mice when they are truly rare, we constructed single-season occupancy models using subsets of our sites where pocket mice captures were low (<5 captures; 30 sites) and where pocket mouse captures were high (≥5 captures; 15 sites). All models included the 50 sites with no pocket mouse detections. We compared detection probabilities resulting from using HA baited with BS to SH traps with PB.

We assessed the effects of grid size on detection probability of pocket mice by calculating the effective trapping area (ETA) of our trapping grids (75×475 m) and traditional grids (90×90 m). To correct for the difference in trap density between the two grid setups we estimated the average minimum and maximum distances moved by pocket mice in a night based on data from the literature [53,54] and our own recapture observations. The minimum nightly distance moved by all individual pocket mice from all species combined was 21.9 m and the maximum was 77.3 m. We buffered the trap grids by those two distances and calculated the area within each buffer [55,56]. We randomly selected an area equal to the ETA of a standard grid from within each of our grids, recorded whether pocket mice were detected in one or more of the traps within that area, and compared the resulting detection of pocket mouse captures to the data from our grids using occupancy modeling [2]. We replicated this process over 1000 bootstrapped standard grid samples and compared the 95% confidence interval (CI) of the resulting distribution of detection probabilities to that from our full-sized grids. We repeated this analysis with increasing grid sizes in order to identify the optimal one in which detection probability is similar, but grid size is smaller, than our full design.

To evaluate the influence of non-target species on detection probability of pocket mice we ran two models. The first occupancy model included deer mouse (*Peromyscus maniculatus*—the most commonly captured non-target species) captures as a covariate. In the second, we calculated the overall number of available trap nights, correcting for closed but empty traps and traps with non-target captures [57]. To correct for the effect of all species (deer mice included) on available trap nights we calculated the proportion of trap nights available for each night at each grid. We then ran an occupancy model with the calculated proportion of available trap nights as a covariate.

## Results

### Captures

We processed a total of 10,987 captures of 5,955 individuals from 21 species during the summers of 2015 and 2016 (Table 1; Fig 1). North American deer mouse, was the most prevalent species captured (72.4% of total captures), followed by Ord’s kangaroo rat (*Dipodomys ordii*; 11.9%), western harvest mouse (*Reithrodontomys megalotis*; 3.5%), olive-backed pocket mouse (*Perognathus fasciatus*; 3.1%), least chipmunk (*Tamias minimus*; 2.0%), sagebrush vole (*Lemmiscus curtatus*; 1.9%), plains harvest mouse (*Reithrodontomys montanus*; 1.3%), prairie vole (*Microtus ochrogaster*; 1.0%), thirteen-lined ground squirrel (*Ictidomys tridecemlineatus*; 0.9%), and northern grasshopper mouse (*Onychomys leucogaster*; 0.7%). The remaining 11 species accounted for only 130 captures (1.2% of total captures; Table 1). Four sites (accounting for 393 captures) were removed from further analysis due to inconsistent baiting methods, yielding a sample size of 95 sites for analysis.

**Table 1:** The number of captures within each species in each trap type.

Species	Common Name	Havahart	Longworth	Sherman	Total
*Peromyscus maniculatus*	North American Deer Mouse	5388	264	2286	7950
*Dipodomys ordii*	Ord’s Kangaroo Rat	758	10	535	1303
*Reithrodontomys megalotis*	Western Harvest Mouse	264	11	107	385
***Perognathus fasciatus***	**Olive-backed Pocket Mouse**	**269**	**7**	**66**	**342**
*Tamias minimus*	Least Chipmunk	133	5	85	223
***Lemmiscus curtatus***	**Sagebrush Vole**	**150**	**1**	**55**	**211**
***Reithrodontomys montanus***	**Plains Harvest Mouse**	**107**	**5**	**31**	**145**
*Microtus ochrogaster*	Prairie Vole	88	1	24	113
*Ictidomys tridecemlineatus*	Thirteen-lined Ground Squirrel	63	4	37	104
*Onychomys leucogaster*	Northern Grasshopper Mouse	50	4	27	81
***Chaetodipus hispidus***	**Hispid Pocket Mouse**	**27**	**0**	**8**	**35**
*Microtus* spp.	Microtus Vole species	18	0	3	21
*Neotoma cinerea*	Bushy-tailed Woodrat	5	0	12	17
***Perognathus mollipilosus***	**Great Basin Pocket Mouse**	**10**	**0**	**3**	**13**
*Urocitellus elegans*	Wyoming Ground Squirrel	1	1	8	10
*Microtus longicaudus*	Long-tailed Vole	7	0	2	9
***Perognathus flavescens***	**Plains Pocket Mouse**	**5**	**0**	**3**	**8**
Unknown	Escaped Individuals	4	0	1	5
*Microtus montanus*	Montane Vole	4	0	0	4
*Sylvilagus* sp.	Cottontail Rabbit species	1	0	2	3
***Perognathus flavus***	**Silky Pocket Mouse**	**2**	**0**	**0**	**2**
*Mustela frenata*	Long-tailed Weasel	1	0	0	1
*Sorex* sp.	Shrew species	0	0	1	1
*Urocitellus* sp.	Ground Squirrel species	0	0	1	1
**Total**	** **	**7355**	**313**	**3297**	**10987**

Ranked from the most captured species to least. Longworth traps had an eighth of the trap effort as Sherman and Havahart traps. Species above the dashed line were included in the analyses for this paper. Species printed in bold type are those classified as being of Greatest Conservation Need in Wyoming [40].

### Trap and bait selection

**Fig 2:**
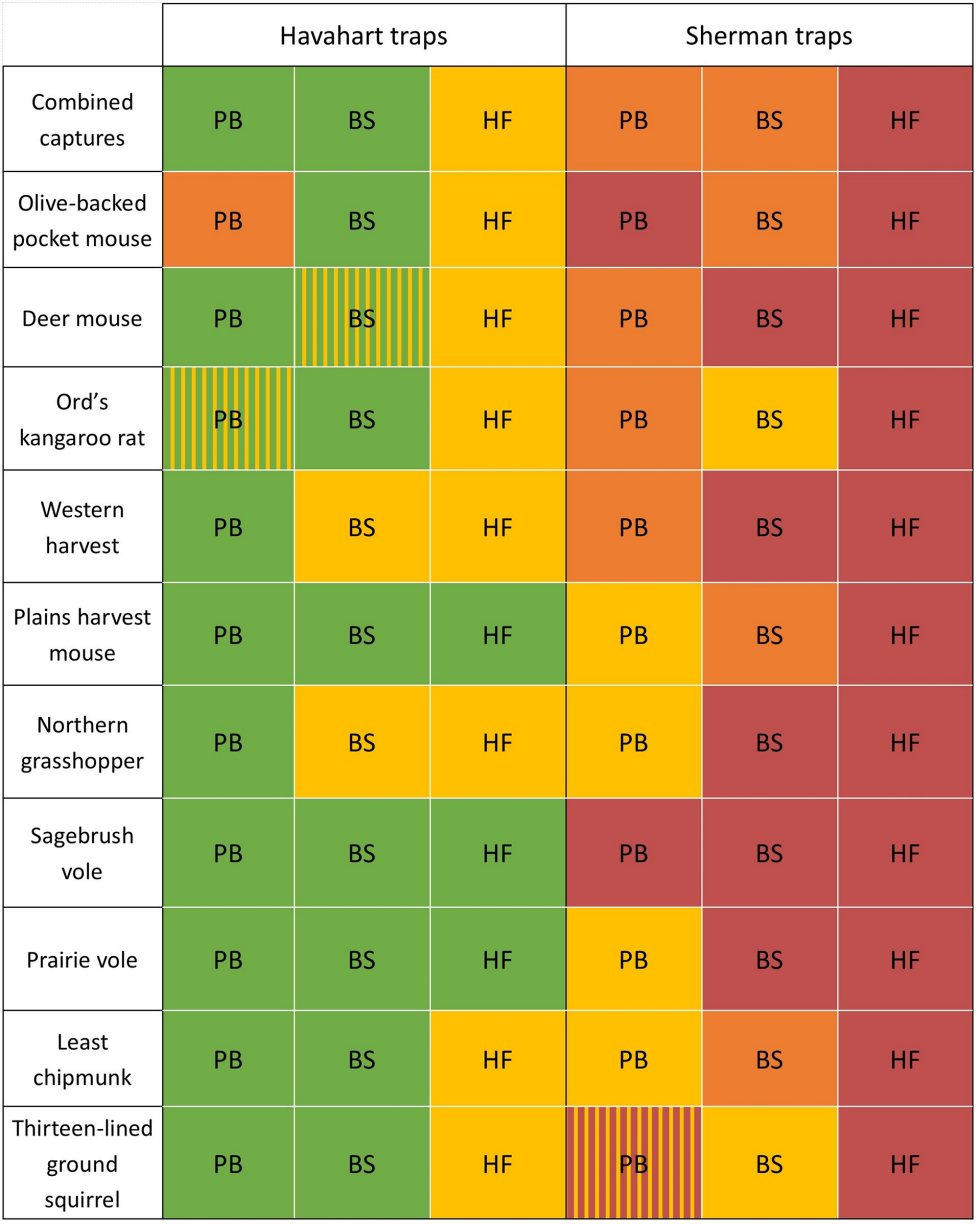
Bait and trap selection by species. Colors represent differences in selection within a species, where trap-bait combinations with the same color are not significantly different from each other. When colors are different, green = strongly positive selection, yellow = weak positive selection, orange = weak avoidance and red = strong avoidance. Bi-color hatching indicates no significant differences from solid colors of the same type. BS = bird seed bait, HF = 3-way horse feed bait, PB = peanut butter bait.

All compositional analyses were significant, indicating selection for specific trap types and baits (Fig 2). HA traps out-performed SH for all species, regardless of bait. Bait selection was less uniform than trap type. Some species selected for BS bait while others selected for PB; HF was generally avoided (Fig 2).

Olive-backed pocket mice showed strong selection for HA traps baited with BS. We observed selection for SH traps using BS to be similar with HA with PB. This shows avoided traps can become more appealing through desirable attractant.

SH traps using HF or PB bait were strongly avoided by pocket mice (Fig 2; S1 Table). The granivorous Ord’s kangaroo rats, relatives of pocket mice, were also trapped most frequently in the HA BS combination (Fig 2; S1 Table) and tended to avoid SH traps with HF bait.

In contrast to olive-backed pocket mice, deer mice were captured most frequently in HA traps with PB bait. Though selection for this combination did not differ from HA traps with BS, it was significantly greater than all other combinations. SH traps with BS bait and SH with HF were least effective at capturing deer mice (Fig 2; S1 Table). Similarly, western harvest mice and northern grasshopper mice showed highest selection for HA traps with PB bait and avoided SH traps with BS or HF bait (Fig 2; S1 Table).

Species whose diets did not match any of the choices we offered (i.e., herbivorous diets), such as sagebrush voles, prairie voles, and plains harvest mice, showed no selection for bait type. Least chipmunks and thirteen-lined ground squirrels selected for HA traps with either BS or PB bait (Fig 2; S1 Table).

**Fig 3:**
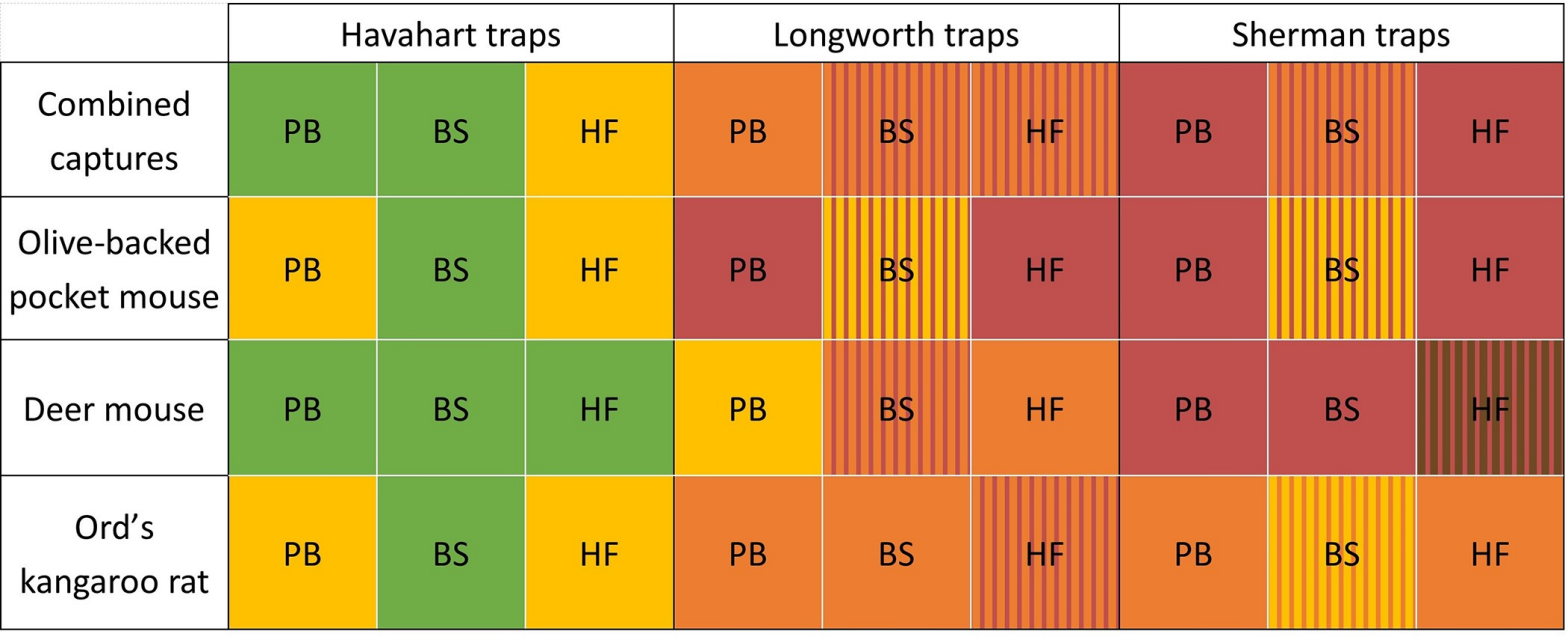
Bait and trap selection by species including Longworth traps. Colors represent differences in selection within a species, where trap-bait combinations with the same color are not significantly different from each other. When colors are different, green = strongly positive selection, yellow = weak positive selection, orange = weak avoidance and red = strong avoidance. Bi-color hatching indicates no significant differences from solid colors of the same type. BS = bird seed bait, HF = 3-way horse feed bait, PB = peanut butter bait.

For the Longworth traps, a subset of captures for combined species and three individual species (deer mice, Ord’s kangaroo rats, and olive-backed pocket mice) revealed that HA traps outperformed the other two types. However, Longworths were significantly selected for compared with SH traps (Fig 3). This overall result was largely driven by deer mice. In contrast, kangaroo rats selected SH trap-combinations over Longworth traps largely because of their size. Olive-backed pocket mice selected HA traps over the other 2 types.

### Detection of pocket mice

Occupancy models including temporal variation as a covariate for detection probability outperformed null models regardless of other trapping variables; detection probability consistently increased by night for all temporal models (Table 2). Detection probabilities for pocket mice on the first night were 3.6 times higher for HA than SH traps (Table 3). This decreased to 3.4, 3.2 and 3.1 times higher on the three subsequent trapping nights (Table 3). Similarly, on the first night, BS bait yielded estimates that were 2.1 and 2.4 times higher than HF and PB baits, respectively. (Table 3). These values declined to 1.5 and 1.7 times higher by the fourth night. On the first night, pocket mouse detection probability for HA traps baited with BS was 11.3 times higher than SH traps with PB bait and 3.1 times higher than SH baited with BS (Table 3). It decreased to 6.6 and 2.1 times higher on the fourth night. This occurred concurrently with overall increasing detection probability for pocket mice suggesting either acclimation to traps or loss of available preferred trap types to other species.

**Table 2:** Values of AICc, delta AIC, AIC weights, and loglikelihood for occupancy models used to determine effect of different trap and bait types on detection probability of pocket mice.

Model	No. of Parameters	AIC	Delta AIC	AIC weights	logLikelihood
**Trap Type**					
Temporal	4	543.48	0	0.99	-267.74
Null	3	552.71	9.23	0.01	-273.36
**Bait Type**					
Temporal	5	761.47	0	1.00	-375.74
Null	4	786.57	25.09	0.00	-389.28
**Combinations**					
Temporal	5	558.59	0	1.00	-274.3
Null	4	572.54	13.94	0.00	-282.27

The data are from 95 sites across Wyoming sagebrush shrubland and prairie grassland trapping during the summers in 2015 and 2016.

**Table 3:** Estimates of detection probability and naïve occupancy for olive-back pocket mice from the best fitting (temporal) model assessing the effects of different trap and bait types.

Detection probability				
	Day 1	Day 2	Day 3	Day 4
**Trap Type**				
Havahart	0.18 (0.12–0.27)	0.24 (0.18–0.32)	0.32 (0.24–0.40)	0.40 (0.30–0.51)
Sherman	0.05 (0.02–0.10)	0.07 (0.03–0.13)	0.10 (0.05–0.18)	0.13 (0.06–0.24)
**Bait Type**				
Bird Seed	0.38 (0.30–0.48)	0.50 (0.42–0.58)	0.61 (0.53–0.68)	0.71 (0.62–0.79)
Horse Feed	0.18 (0.12–0.26)	0.26 (0.20–0.33)	0.35 (0.28–0.44)	0.46 (0.36–0.57)
Peanut Butter	0.16 (0.11–0.23)	0.23 (0.17–0.30)	0.32 (0.25–0.40)	0.43 (0.33–0.53)
**Combination**				
HA_BS	0.34 (0.25–0.45)	0.45 (0.37–0.53)	0.56 (0.47–0.64)	0.66 (0.54–0.76)
SH_BS	0.11 (0.07–0.18)	0.16 (0.11–0.23)	0.23 (0.16–0.30)	0.31 (0.22–0.42)
SH_PB	0.03 (0.01–0.06)	0.04 (0.02–0.08)	0.07 (0.03–0.12)	0.10 (0.05–0.18)

The data are from 95 sites across Wyoming sagebrush shrubland and prairie grassland trapping during the summers in 2015 and 2016. HA = Havahart traps, SH = Sherman traps, BS = bird seed bait, HF = 3-way horse feed bait, PB = peanut butter bait.

The detection probabilities for HA traps with BS bait were 1.8 times higher for sites where pocket mouse captures were high (mean = 0.142, 95% CI = 0.050–0.341) compared to when they were low (mean = 0.079, 95% CI = 0.030–0.190). In contrast, detection probability for SH baited with PB was essentially zero in either case (0.003, 95% CI = 0.000–0.028 and 0.007, 95% CI = 0.001–0.046, respectively), suggesting that the likelihood of detecting pocket mice with standard protocols is severely reduced, regardless of their abundance.

### Grid size

**Fig 4:**
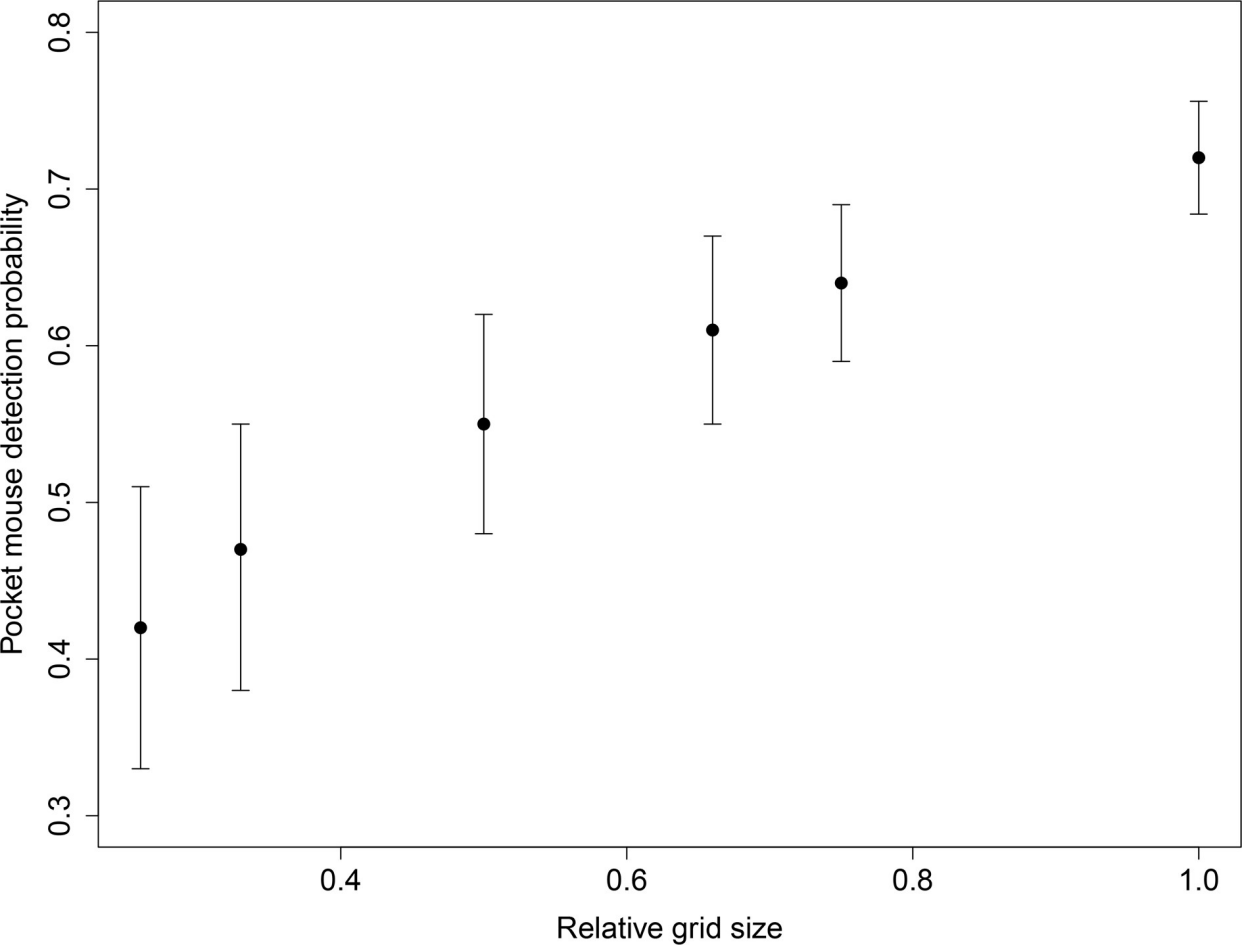
Effect of grid size on olive-backed pocket mouse detection probability. Detection probability for each grid size is in black with 95% confidence interval. Grid sizes used were 0.26, 0.33, 0.50, 0.66 and 0.75 of the grid used in our study.

The estimated ETA for our grid design was 4.86 ha– 8.41 ha, depending on the calculated movement distances of pocket mice. The ETA for a simulated standard grid was 1.25–2.80 ha, or 25.7–33.3% of our design. The overall estimated detection probability for our full-sized grid was 0.72 (95% CI = 0.69–0.75), which was 1.5 to 1.7 times higher than the distribution of detection probabilities from our bootstrapped standard-sized grids (0.42, 95% CI = 0.33–0.51 for 25.7% grids and 0.47, 95% CI = 0.39–0.55 for 33.3% grids; Fig 4). Our evaluation of intermediate grid sizes revealed that the ETA of trapping grids would have to be at least 75% of our full-sized grid before the estimated detection probability was comparable (Fig 4).

### Non-target species

**Fig 5:**
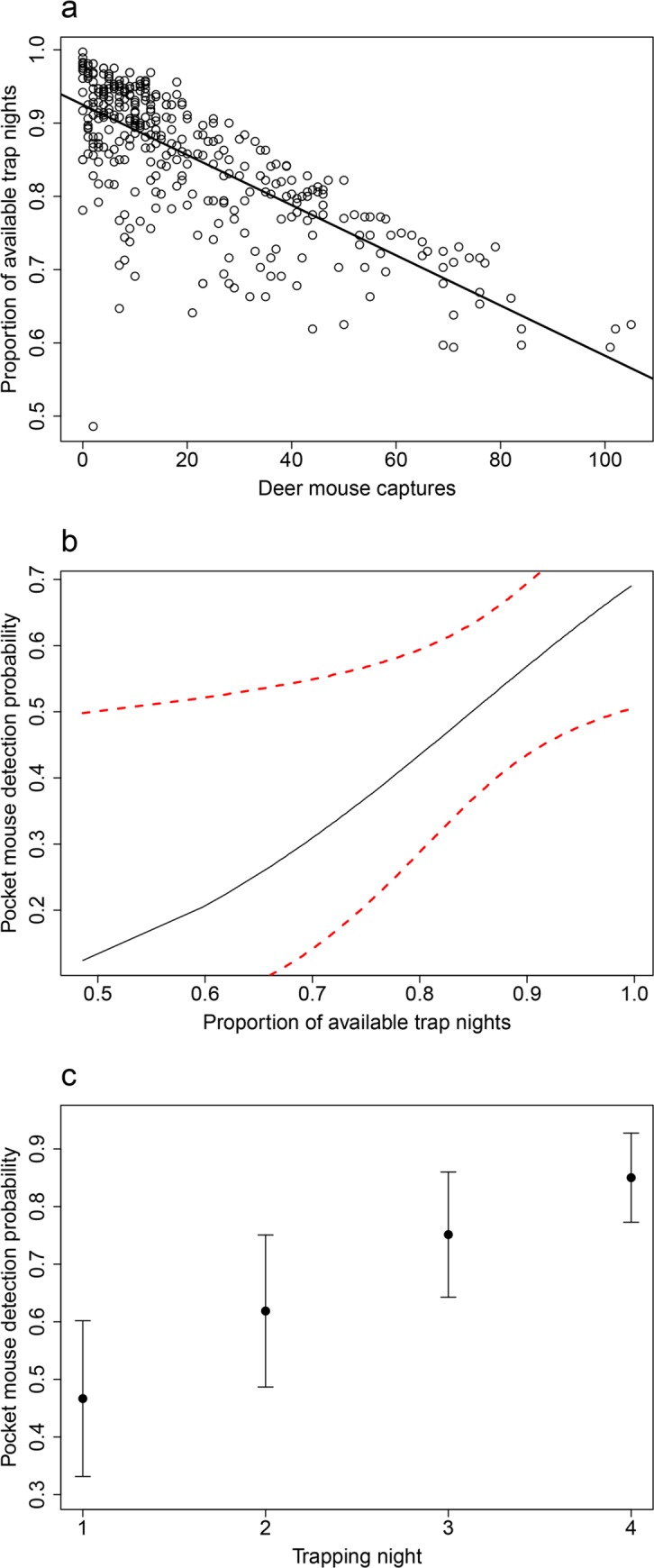
The effect of deer mouse abundance on pocket mouse detection. A. Proportion of trap nights available relative to deer mouse captures showing the negative effect of these small mammals on trap availability for other species. B. The pocket mouse detection probability on day 1 relative to the proportion of trap nights available, corrected for closed but empty traps and non-pocket mouse captures, with 95% confidence intervals (dashed red). C. Pocket mouse detection probability by trapping night showing the temporal increase in detection given availability of traps.

Without accounting for disturbed traps or captures of non-target species, the available trap-nights for capturing pocket mice in a single night in each grid ranged from 160 to 180. After applying the correction, available trap-nights ranged from 88.0 to 179.5 (mean = 146.7, 95% CI = 124.4–169), which equated to a proportion of available trap-nights of 0.48 to 0.99 (mean = 0.85, 95% CI = 0.75–0.95). Although the effect of deer mouse captures did not have a direct effect on the detection of pocket mice (*p* = 0.15), it had a significant influence on the proportion of available trap nights (*p* < 0.01, Fig 5A). As the proportion of available trap nights increased, pocket mouse detection probability also increased for each of the sampling nights (*p* < 0.02, Fig 5B and 5C).

## Discussion

Our results suggest that Havahart traps, especially when baited with bird seed, are more effective at increasing capture success, and thus detection probability, of pocket mice compared with the standard trap and bait combination of Sherman traps and peanut butter-oats mix. Grid size further affected pocket mouse detection. These results indicate that using standard small mammal trapping methods (i.e., a 10×10 grid of Shermans spaced 10 m apart and baited with peanut butter and oats) will likely result in negative bias in modeling occupancy of pocket mice and other rare species.

The success of Havaharts can be attributed to several of their features. First, Havaharts have doors on both ends of the trap which doubles the chance a small mammal will enter it. In addition, the mesh sides of Havaharts also reduce their appearance as an enclosed space while allowing the aromas of the bait to permeate over a larger area. Finally, the triggers of Havaharts are notoriously sensitive, so the traps may be activated more easily by movements of exploratory animals. We were unable to assess whether trigger sensitivity was the driving factor behind Havahart success, because animals of all body mass were trapped in all three trap types.

Unfortunately, while Havaharts appear to be more effective, using them is problematic for several reasons: 1. These traps do not collapse which makes them more difficult to carry; 2. They require more effort and time to set properly; and 3. Animals captured in them are more exposed to inclement conditions. Of the 5,956 individuals captured in this project, 221 died, with 48% of the mortalities caused by exposure; an additional 65 small mammals experienced trap-related injuries. Most exposure-related mortalities (91%) occurred in Havaharts. In contrast, only 30% of injuries (broken tails–especially in kangaroo rats, broken feet–mostly in deer mice, etc.) occurred in these traps. Given their success in capturing small mammals, learning to quickly and efficiently set these traps, as well as refining methods to reduce exposure of animals to inclement conditions (such as placing a wooden coverboard or tarp over the trap), will likely be worth the effort as they substantially increase the capture probability of difficult to detect species.

For pocket mice, it appears that using bird seeds as bait also increases detection probability, even when using Shermans. It is not a surprise that pocket mice select for bird seed considering their granivorous diet. A number of studies of small mammals in desert and shrubland ecosystems used bird seed as bait based on the natural history of the dominant species in those regions (*Dipodomys* and *Perognathus*; [21,31,37–39]. Based on our results, we recommend that this approach be considered whenever the target species are known to specialize on specific diet items. We noted that sagebrush and prairie voles showed little preference for the bait types we used, most likely because they largely feed on green plant material such as grass and sedge stalks and sagebrush leaves [43,44]. Studies targeting vole species may need to adjust bait type to alfalfa pellets or fresh hay to increase trapping success of these mammals. Additionally, while the carnivorous grasshopper mice showed strong selection for peanut butter they may be better attracted to traps with bait derived from insects or meat [58,59], which would reduce by-catch of other small mammal species that do not consume such foods. By extension, if the goal of the study is to sample the entire small mammal community, it may be more effective to use multiple bait-types to appeal to a larger range of species. More specifically, studies investigating community composition could yield biased results if they use only one bait type that is preferred by some species and avoided by others, because such a design would differentially affect detectability of those species.

Trapping small mammals poses unique challenges compared to trapping other taxa because the number of traps is finite and unwanted by-catch will inherently reduce trap availability. Captures of non-target species (the most prevalent of which was deer mouse at 72% of all captures) significantly impacted traps available to capture pocket mice, which resulted in decreased detection probability (Fig 5; [57]). It is likely that by offering two or three traps at each station and including multiple types of bait, some of which were more preferable to non-target species, we saturated the trapped area with detectors and increased the likelihood that traps with bait preferred by our target species were available to capture those species. This may make trapping more equivalent to methods like electrofishing [60], where the capture of non-target species does not prevent the detection of rare or difficult to detect fish species. Similarly, detecting rare species of bats or birds using mist netting is not affected by by-catch; rather captures of species are more dependent on the net location and species-specific traits [61,62]. However, when trapping small mammals, researchers have few options for eliminating by-catch, which means that abundant and/or trap-happy species, such as the deer mouse, have the potential to ‘flood’ traps and prevent the capture of other species [36,63]. Having multiple traps at a station and altering bait and trap types may reduce the effects of by-catch when targeting specific species.

The design of our trapping grid further increased detection probabilities for pocket mice when compared to the standard trapping grid. Detection probability for pocket mice was significantly lower for the 25.7% and 33.3% sized grids relative to our full one. The difference was not significant for larger grids. This suggests that adjusting the size of the our grid down (e.g., 20 m spacing or 4×15 trap stations) would facilitate the effective detection of pocket mice or other rare species without the substantial effort that was required to trap the 3.56 ha grids used in our study. Overall, our results suggest that if researchers intend to use Sherman traps, results could still be meaningfully improved by increasing grid size and selecting proper bait.

By altering trap type, bait type and grid size we were able to increase our detection probability in occupancy models for pocket mice up to 11 times that resulting from standard trapping methods used on small mammals. In contrast with other studies, which improved detectability by employing two different survey methods (e.g., surveys and eDNA, [7,9]); camera traps and live trapping, [64]), we were able to increase detection of pocket mice by modifying a single method to achieve improved results with roughly similar effort and cost as the standard method of trapping. Given these benefits, we believe our methods, particularly using multiple bait types, increasing the number of Havahart traps, and slightly increasing grid size, should be adopted in other studies seeking to increase detectability of rare small mammals.

## Conclusion

Our results suggest that simple modifications to the standard small mammal trapping-methods could improve their effectiveness, thereby increasing the robustness of conclusions from studies targeting rare and difficult to detect species with differing diet preferences. For pocket mice, this increased level of detection has the potential to improve our knowledge of their ecology, habitat suitability and occupancy trends across their range. In addition, our protocols should be considered by researchers attempting to assess community and ecosystem dynamics. Failure to detect some members of the community may yield incorrect estimates of community composition, species richness and interactions, niche size and overlap, as well as energy flow in ecosystems [65,66]. For example, whole community energy consumption will be relatively constant when the abundance of competing species alternate [66]. The effects of low detection of one of these interacting species, even when they are at high densities (as would be the case for pocket mice trapped with peanut butter and Sherman traps), could result in miscalculation of whole community energy consumption. In turn, failure to properly describe community interactions will reduce the credibility of management decisions for species and ecosystems in the face of a rapidly changing environment.

## Supporting information

S1 Table Compositional analysis results for combined captures and the ten rodent species with the highest number of captures. The matric contains the mean difference between used and available log-ratios. Positive values (selection) are in bold. An asterisk after the value represents significance. (HA = Havahart, SH = Sherman, BS = Bird Seed, HF = Horse Feed, PB = Peanut Butter)
(DOCX)
