# Supplementary material for: It’s a trap: Optimizing detection of rare small mammals
Source: PLoS One. 2019 Mar 5;14(3):e0213201. doi: 10.1371/journal.pone.0213201 (PMC6400386; doi:10.1371/journal.pone.0213201)
Supplement: S1 Table — The matric contains the mean difference between used and available log-ratios. Positive values (selection) are in bold. An asterisk after the value represents significance. (HA = Havahart, SH = Sherman, BS = Bird Seed, HF = Horse Feed, PB = Peanut Butter) (DOCX) [file pone.0213201.s001.docx]

**S1 Table**

| A. All species |  |  |  |  |  |  |  | B. Deer mouse |  |  |  |  |  |  |
| --- | --- | --- | --- | --- | --- | --- | --- | --- | --- | --- | --- | --- | --- | --- |
|  | HA_BS | HA_HF | HA_PB | SH_BS | SH_HF | SH_PB |  |  | HA_BS | HA_HF | HA_PB | SH_BS | SH_HF | SH_PB |
| HA_BS | 0 | **0.29** | -0.05 | **1.58** | **2.16** | **1.40** |  | HA_BS | 0 | **0.18** | -0.12 | **2.02*** | **2.39*** | **1.45*** |
| HA_HF | -0.29 | 0 | -0.34 | **1.28** | **1.87** | **1.11** |  | HA_HF | -0.18 | 0 | -0.30 | **1.84*** | **2.22*** | **1.27*** |
| HA_PB | **0.05** | **0.34** | 0 | **1.62** | **2.21** | **1.44** |  | HA_PB | **0.12** | **0.30** | 0 | **2.14*** | **2.52*** | **1.57*** |
| SH_BS | -1.58 | -1.28 | -1.62 | 0 | **0.59** | -0.18 |  | SH_BS | -2.02* | -1.84* | -2.14* | 0 | **0.37** | -0.57* |
| SH_HF | -2.16 | -1.87 | -2.21 | -0.59 | 0 | -0.76 |  | SH_HF | -2.39* | -2.22* | -2.52* | -0.37 | 0 | -0.95* |
| SH_PB | -1.40 | -1.11 | -1.44 | **0.18** | **0.76** | 0 |  | SH_PB | -1.45* | -1.27* | -1.57* | **0.57*** | **0.95*** | 0 |
|  |  |  |  |  |  |  |  |  |  |  |  |  |  |  |
| C. Ord's kangaroo rat |  |  |  |  |  |  |  | D. Western harvest mouse |  |  |  |  |  |  |
|  | HA_BS | HA_HF | HA_PB | SH_BS | SH_HF | SH_PB |  |  | HA_BS | HA_HF | HA_PB | SH_BS | SH_HF | SH_PB |
| HA_BS | 0 | **0.49*** | **0.19** | **0.79*** | **1.40*** | **1.12*** |  | HA_BS | 0 | **0.02** | -0.49* | **1.19*** | **1.17*** | **0.55*** |
| HA_HF | -0.49* | 0 | -0.30 | **0.30** | **0.91*** | **0.63*** |  | HA_HF | -0.02 | 0 | -0.50* | **1.18*** | **1.16*** | **0.53*** |
| HA_PB | -0.19 | **0.30** | 0 | **0.60*** | **1.21*** | **0.93*** |  | HA_PB | **0.49*** | **0.50*** | 0 | **1.68*** | **1.66*** | **1.03*** |
| SH_BS | -0.79* | -0.30 | -0.60* | 0 | **0.61*** | **0.33*** |  | SH_BS | -1.19* | -1.18* | -1.68* | 0 | -0.02 | -0.64* |
| SH_HF | -1.40* | -0.91* | -1.21* | -0.61* | 0 | -0.28* |  | SH_HF | -1.17* | -1.16* | -1.66* | **0.02** | 0 | -0.62* |
| SH_PB | -1.12* | -0.63* | -0.93* | -0.33* | **0.28*** | 0 |  | SH_PB | -0.55* | -0.53* | -1.03* | **0.64*** | **0.62*** | 0 |
|  |  |  |  |  |  |  |  |  |  |  |  |  |  |  |
| E. Olive-backed pocket mouse |  |  |  |  |  |  |  | F. Least chipmunk |  |  |  |  |  |  |
|  | HA_BS | HA_HF | HA_PB | SH_BS | SH_HF | SH_PB |  |  | HA_BS | HA_HF | HA_PB | SH_BS | SH_HF | SH_PB |
| HA_BS | 0 | **0.76*** | **1.22*** | **1.51*** | **2.18*** | **2.14*** |  | HA_BS | 0 | **0.48*** | **0.03** | **0.92*** | **1.47*** | **0.55*** |
| HA_HF | -0.76* | 0 | **0.47*** | **0.75*** | **1.42*** | **1.38*** |  | HA_HF | -0.48* | 0 | -0.46* | **0.43*** | **0.98*** | **0.07** |
| HA_PB | -1.22* | -0.47* | 0 | **0.28** | **0.96*** | **0.91*** |  | HA_PB | -0.03 | **0.46*** | 0 | **0.89*** | **1.44*** | **0.53*** |
| SH_BS | -1.51* | -0.75* | -0.28 | 0 | **0.67*** | **0.63*** |  | SH_BS | -0.92* | -0.43* | -0.89* | 0 | **0.55*** | -0.36* |
| SH_HF | -2.18* | -1.42* | -0.96* | -0.67* | 0 | -0.04 |  | SH_HF | -1.47* | -0.98* | -1.44* | -0.55* | 0 | -0.91* |
| SH_PB | -2.14* | -1.38* | -0.91* | -0.63* | **0.04** | 0 |  | SH_PB | -0.55* | -0.07 | -0.53* | **0.36*** | **0.91*** | 0 |
|  |  |  |  |  |  |  |  |  |  |  |  |  |  |  |
| G. Sagebrush vole |  |  |  |  |  |  |  | H. Plains harvest mouse |  |  |  |  |  |  |
|  | HA_BS | HA_HF | HA_PB | SH_BS | SH_HF | SH_PB |  |  | HA_BS | HA_HF | HA_PB | SH_BS | SH_HF | SH_PB |
| HA_BS | 0 | -0.10 | **0.11** | **1.02*** | **1.14*** | **1.07*** |  | HA_BS | 0 | **0.05** | -0.08 | **0.76*** | **0.96*** | **0.66*** |
| HA_HF | **0.10** | 0 | **0.20** | **1.11*** | **1.24*** | **1.17*** |  | HA_HF | -0.05 | 0 | -0.13 | **0.71*** | **0.91*** | **0.61*** |
| HA_PB | -0.11 | -0.20 | 0 | **0.91*** | **1.03*** | **0.96*** |  | HA_PB | **0.08** | **0.13** | 0 | **0.84*** | **1.04*** | **0.74*** |
| SH_BS | -1.02* | -1.11* | -0.91* | 0 | **0.13** | **0.06** |  | SH_BS | -0.76* | -0.71* | -0.84* | 0 | **0.20*** | -0.10* |
| SH_HF | -1.14* | -1.24* | -1.03* | -0.13 | 0 | -0.07 |  | SH_HF | -0.96* | -0.91* | -1.04* | -0.20* | 0 | -0.30* |
| SH_PB | -1.07* | -1.17* | -0.96* | -0.06 | **0.07** | 0 |  | SH_PB | -0.66* | -0.61* | -0.74* | **0.10*** | **0.30*** | 0 |
|  |  |  |  |  |  |  |  |  |  |  |  |  |  |  |
| I. Prairie vole |  |  |  |  |  |  |  | J. Thirteen-lined ground squirrel |  |  |  |  |  |  |
|  | HA_BS | HA_HF | HA_PB | SH_BS | SH_HF | SH_PB |  |  | HA_BS | HA_HF | HA_PB | SH_BS | SH_HF | SH_PB |
| HA_BS | 0 | 0.00 | -0.01 | **1.02*** | **1.06*** | **0.81*** |  | HA_BS | 0 | **0.72*** | **0.13** | **0.74*** | **1.17*** | **0.92*** |
| HA_HF | 0.00 | 0 | -0.01 | **1.02*** | **1.06*** | **0.81*** |  | HA_HF | -0.72* | 0 | -0.58* | **0.02** | **0.45*** | **0.20** |
| HA_PB | **0.01** | **0.01** | 0 | **1.03*** | **1.07*** | **0.82*** |  | HA_PB | -0.13 | **0.58*** | 0 | **0.61*** | **1.04*** | **0.78*** |
| SH_BS | -1.02* | -1.02* | -1.03* | 0 | **0.04** | -0.21* |  | SH_BS | -0.74* | -0.02 | -0.61* | 0 | **0.43*** | **0.18** |
| SH_HF | -1.06* | -1.06* | -1.07* | -0.04 | 0 | -0.25* |  | SH_HF | -1.17* | -0.45* | -1.04* | -0.43* | 0 | -0.25 |
| SH_PB | -0.81* | -0.81* | -0.82* | **0.21*** | **0.25*** | 0 |  | SH_PB | -0.92* | -0.20 | -0.78* | -0.18 | **0.25** | 0 |
|  |  |  |  |  |  |  |  |  |  |  |  |  |  |  |
| K. Northern grasshopper mouse |  |  |  |  |  |  |  |  |  |  |  |  |  |  |
|  | HA_BS | HA_HF | HA_PB | SH_BS | SH_HF | SH_PB |  |  |  |  |  |  |  |  |
| HA_BS | 0 | -0.02 | -0.43* | **0.81*** | **0.81*** | **0.27** |  |  |  |  |  |  |  |  |
| HA_HF | **0.02** | 0 | -0.41* | **0.83*** | **0.83*** | **0.29** |  |  |  |  |  |  |  |  |
| HA_PB | **0.43*** | **0.41*** | 0 | **1.24*** | **1.24*** | **0.70*** |  |  |  |  |  |  |  |  |
| SH_BS | -0.81* | -0.83* | -1.24* | 0 | 0.00 | -0.54* |  |  |  |  |  |  |  |  |
| SH_HF | -0.81* | -0.83* | -1.24* | 0.00 | 0 | -0.54* |  |  |  |  |  |  |  |  |
| SH_PB | -0.27 | -0.29 | -0.70* | **0.54*** | **0.54*** | 0 |  |  |  |  |  |  |  |  |
